# Comparison of statistical approaches to rare variant analysis for quantitative traits

**DOI:** 10.1186/1753-6561-5-S9-S113

**Published:** 2011-11-29

**Authors:** Han Chen, Audrey E Hendricks, Yansong Cheng, Adrienne L Cupples, Josée Dupuis, Ching-Ti Liu

**Affiliations:** 1 Department of Biostatistics, Boston University School of Public Health, Boston, MA 02118, USA

## Abstract

With recent advances in technology, deep sequencing data will be widely used to further the understanding of genetic influence on traits of interest. Therefore not only common variants but also rare variants need to be better used to exploit the new information provided by deep sequencing data. Recently, statistical approaches for analyzing rare variants in genetic association studies have been proposed, but many of them were designed only for dichotomous outcomes. We compare the type I error and power of several statistical approaches applicable to quantitative traits for collapsing and analyzing rare variant data within a defined gene region. In addition to comparing methods that consider only rare variants, such as indicator, count, and data-adaptive collapsing methods, we also compare methods that incorporate the analysis of common variants along with rare variants, such as CMC and LASSO regression. We find that the three methods used to collapse rare variants perform similarly in this simulation setting where all risk variants were simulated to have effects in the same direction. Further, we find that incorporating common variants is beneficial and using a LASSO regression to choose which common variants to include is most useful when there is are few common risk variants compared to the total number of risk variants.

## Background

Genome-wide association studies have successfully identified many novel common risk alleles associated with complex traits. Yet these common genetic variants typically have small effect sizes and explain only a small portion of genetic variation for a certain trait. With the advances in whole-genome sequencing technology, data on rare variants have become increasingly available, and many investigators hope that rare variants will enhance our understanding of the biological mechanisms of human diseases and traits. Recently, novel statistical approaches have been proposed to assess the association between traits and rare variants, sometimes including and sometimes excluding nearby common variants. However, many of these methods were developed in a case-control framework and are not applicable to quantitative traits. In this paper, we implement several recently published approaches that can be applied to quantitative traits and compare their type I error and power using the Genetic Analysis Workshop 17 (GAW17) data set.

Traditionally, investigators have evaluated genetic trait association by examining a trait’s pattern among the genotypes of single-nucleotide polymorphisms (SNPs), with each SNP being analyzed independently of the other SNPs. Often a regression model is used to account for potential confounding covariates. However, this approach is not adequate for rare variants because the power is directly related to the minor allele frequency (MAF) and is especially decreased when the MAF is low. An alternative to testing each rare variant separately is to combine information across variants in a defined gene region. Intuitively, we can collapse information across different loci for rare variants by dichotomizing the existence of at least one rare variant (indicator method) or by counting the number of minor alleles of the rare variants (count method). These summary measures can then be assessed for association with the trait of interest using a regression or other statistical framework.

To analyze rare and common variants simultaneously, Li and Leal [1] proposed a new approach, the combined multivariate and collapsing (CMC) method, to detect association between a predefined functional unit or region and a trait. In 2010, Han and Pan [2] proposed a data-adaptive sum test to incorporate each rare variant’s direction of association (data-adaptive method). More recently, Morris and Zeggini [3] described how to use a rare allele proportion in a linear regression framework. Their proportion method is equivalent to using the count method when there are no missing genetic data.

One drawback to the CMC method is that the approach incorporates all common variants in the test statistic. Although this certainly retains any markers that are truly associated, including all common variants likely also retains many falsely associated markers. To balance the inclusion of noise and true signals, we modify the CMC analysis by first using least absolute shrinkage and selection operator (LASSO) regression [4] to select common variants to include in the multivariate statistic.

In this study, we compare the power and type I error of three methods (indicator, count, and data-adaptive) for collapsing rare variants in a gene region across three strategies (no common variants, CMC, and LASSO) to account for the common variants in the gene region.

## Methods

We use the simulated GAW17 data to calculate type I error and power. Genetic data from the 1000 Genomes Project was used to represent exome sequencing in the GAW17 data set. The sample consists of 697 unrelated subjects from seven populations and includes the original sex and age of each subject. Smoking status and traits are simulated across various association scenarios for 200 replicates. We calculate type I error for each method using quantitative trait Q4, a trait with no association to any of the genotypes. We estimate the null distribution for each method by pooling the results for trait Q4 from all gene regions and across all 200 replicates and derive an empirical significance threshold for each method from the empirical null distribution. We then calculate power for the nine genes associated with quantitative trait Q1 using the corresponding empirical significance threshold for each method. We define rare variants as SNPs with a MAF less than 0.005, 0.01, or 0.05. SNPs are assigned to a gene region using the gene assignment in the GAW17 SNP information file.

To evaluate the association between Q1 and each gene region, we calculate an *F* statistic by comparing the regression model containing genetic variables to the model without genetic variables. In each case, we adjust for sex, age, smoking status, and population group.

### Collapsing rare variants

For the indicator method, we assign a dichotomous code to genes with rare variants by creating an indicator variable for each of those genes. The rare variant score is set to 1 if a subject has one or more rare variants within the gene region and 0 if a subject has no rare variants within the gene region.

For the count method, we use a count code for each gene with rare variants. Thus for each gene with rare variants and for each subject, we count the total number of minor alleles for each gene region’s rare variants and use the count as the rare variant score.

Finally, for the data-adaptive sum test, we implement the method first introduced by Han and Pan [2]. For each phenotype simulation replicate, we code each rare variant additively using the number of minor alleles and then fit a linear regression model using Q1 or Q4 as the outcome and the covariates and the additively coded rare variant as the predictors. If the coefficient of the rare variant is less than 0 and the p-value is less than or equal to 0.1, we flip the variant coding; in other words, we count the number of major alleles. For each gene with rare variants, we sum all coded variants within the gene region to get the rare variant score. Our implementation of the data-adaptive method is nearly identical to Han and Pan’s method. However, Han and Pan used permutation to approximate the null distribution of their statistic and hence to control for the inflated type I error that occurs when the same data are used to flip the allele coding and then to test for association with the rare variant score. Performing permutation for all 200 simulation replicates was computationally infeasible because of time constraints. Therefore we estimate the null distribution using Q4.

### Inclusion of common variants

We use three methods for collapsing rare variants to test the association between the rare variant scores and quantitative traits without using common variants. We also perform analyses including both rare and common variants. For the two methods that include common variants, we apply the three methods for collapsing rare variants and thus arrive at six approaches to jointly test common and rare variants.

For the CMC method, and for genes with rare variants only, we run a linear regression model using Q1 or Q4 as the outcome and the rare variant score and the previously listed covariates as the predictors. For genes with both rare variants and common variants we include each of the common variants in the model using an additive genetic model. Finally, we calculate the *F* statistic as previously described to evaluate the genetic effect.

For the LASSO method, we run a LASSO regression using the glmnet package in R [5,6] for each gene region, forcing in all the covariates as well as the rare variant score. Thus we use the LASSO only to select which common variants to retain in the model. We use 10-fold cross validation (CV) to find the shrinkage parameter (*λ*) with the minimum CV error. To promote smaller models, we choose the largest *λ* within 1 unit of standard error from the minimum *λ* for our final LASSO model. The covariates, rare variant score, and common variants remaining in the model after LASSO selection are then taken to a linear regression model, and genetic association is tested using the *F* statistic, as previously described.

## Results

As shown in Table 1, for the methods that incorporated common variants, the data-adaptive approach had slightly elevated type I error rates compared to the indicator and count methods, and the LASSO method had inflated type I error rates, whereas the CMC method retained type I error rates close to the chosen significance level. For the LASSO method, the type I error decreased as the MAF cutoffs increased, whereas the type I error was consistent across MAF cutoffs for the CMC method and also when common variants were not included.

**Table 1:** Type I error (5% significance level)

MAF cutoff	Collapsing method (no common variants)			CMC method			LASSO method		
	
	Indicator	Count	Data-adaptive	Indicator	Count	Data-adaptive	Indicator	Count	Data-adaptive
0.005	5.1	5.1	7.8	5.2	5.2	6.2	7.9	7.9	10.5
0.01	5.1	5.2	7.5	5.1	5.2	6.3	6.9	6.9	9.2
0.05	5.0	5.1	7.0	5.0	5.1	6.3	5.7	5.7	7.5

**Figure 1:**
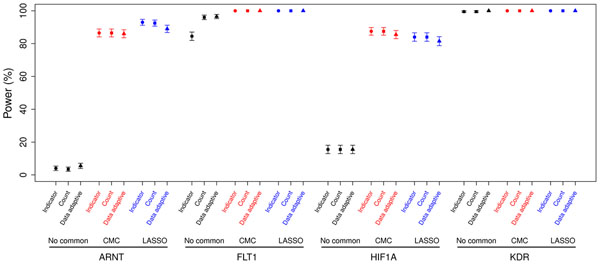
**Power to detect genes with common variants**. A MAF threshold of 0.01 is used to define rare variants.

**Figure 2:**
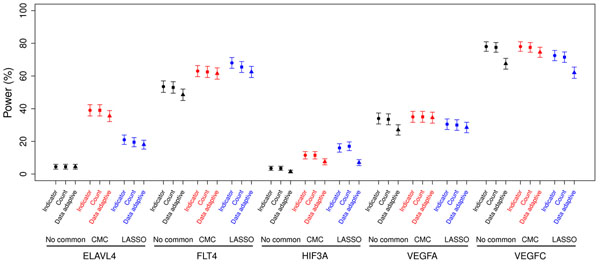
**Power to detect genes without common variants.** A MAF threshold of 0.01 is used to define rare variants.

For the gene regions with causal common variants (Figure 1), including common variants in the model using the CMC or the LASSO method produced higher power compared with not including the common variants in the model. For most of these gene regions, there was no large difference in the power of the CMC and LASSO methods. Including common variants in the model also produced a slight increase in power across most gene regions not containing causal common variants (Figure 2). The three methods for collapsing rare variants (indicator, count, and data-adaptive) had similar power for each gene region and the methods that included common variants. For a few genes, the data-adaptive method had slightly lower power compared to the indicator and count methods. Finally, the power across MAF cutoffs differed within some gene regions and was consistent across others (data not shown).

## Discussion

Unless otherwise indicated, we focus our discussion on the results using the MAF cutoff of 0.01 to define rare variants.

We expected that each method would perform well under different circumstances. For the collapsing of rare variants, we expected the indicator and count methods to perform similarly in many situations because most individuals have only one or a few rare causal variants in each gene region. The count method outperforms the indicator method only when there is non-negligible probability of subjects having multiple rare variants, each of which has a marginal association with the trait in the same direction. The increase in power for the count method compared to the indicator method can be seen in *FLT1*, which was simulated to have 8 out of 25 causal rare variants, but the increase in power is not seen in *KDR*, which was simulated to have 8 out of 14 causal rare variants, because all methods have 100% power for *KDR* (Table 2 & Figure 1). We expected the data-adaptive method to outperform the count and indicator methods only when fairly equal numbers of rare variants in a gene region have opposite effects. Because all the causal variants were simulated to have effects in the same direction, we did not observe the data-adaptive method outperforming the count or indicator method. However, it is worth noting that the power of the data-adaptive method is not highly diminished. Thus the data-adaptive method may be the better choice for real data when we expect some genes to contain rare variants with both detrimental and beneficial effects. More research is needed using simulations with effects in both directions.

**Table 2:** Proportion of rare and common causal SNPs per risk gene

Gene	MAF cutoff (number of causal SNPs/number of total SNPs)					
	
	0.005		0.01		0.05	
	
	Rare	Common	Rare	Common	Rare	Common
*ARNT*	4/14	1/4	4/15	1/3	5/17	0/1
*ELAVL4*	2/7	0/3	2/7	0/3	2/8	0/2
*FLT1*	8/24	3/11	8/25	3/10	10/32	1/3
*FLT4*	2/8	0/2	2/8	0/2	2/10	0/0
*HIF1A*	3/6	1/2	3/7	1/1	4/8	0/0
*HIF3A*	3/13	0/8	3/15	0/6	3/17	0/4
*KDR*	8/14	2/2	8/14	2/2	9/15	1/1
*VEGFA*	1/5	0/1	1/5	0/1	1/6	0/0
*VEGFC*	1/1	0/0	1/1	0/0	1/1	0/0

In regards to incorporating common variants, we expected that the methods that incorporated common variants (CMC and LASSO) would perform well when common variants in a gene region explained a relatively moderate to high proportion of the gene region association with the trait compared to rare variants. This is seen most readily with the genes *ARNT* and *HIF1A*. Although difficult to detect because of the high power across all methods, a slight increase is also seen for *FLT1*.

Interestingly, incorporating common variants also increased the power for genes that did not contain any causal common variants, albeit to a lesser extent than seen in the genes that did have causal common variants. This slight increase in power seen for *ELAVL4*, *FLT4*, and *HIF3A* is likely due to moderate linkage disequilibrium between common variants and causal rare variants.

We expected the LASSO method to outperform the CMC method when the ratio of common variants associated with the trait to the total number of common variants was low. Many common variants not associated with the outcome cause noise in the CMC method, whereas LASSO regression is able to filter out some of the noise, leading to a more powerful result. Out of the four risk genes that contain common risk variants, only *ARNT* and *FLT1* have a ratio less than 1 (1/3 and 3/10, respectively). For *FLT1*, the simulated effect is too strong to differentiate between methods (maximum *p*-value for either method is less than 1 × 10^−14^). However, in *ARNT* we do see a slight increase in power for the LASSO regression, as expected. The increase in power for the LASSO method to detect *ARNT* is small, probably because the ratio of causal common SNPs to total common SNPs is moderate, indicating that not much noise (only two SNPs) can be removed from the regression by using selection on the common SNPs. As the ratio decreases, we would expect to see a sizable increase in power for the LASSO method. Research using different simulation designs is needed to verify this expectation. When the ratio of common variants associated with the outcome to the total number of common variants is large, we expect the CMC and LASSO methods to perform similarly, as seen with the genes *HIF1A* and *KDR*, each of which has a ratio of 1.

We also looked at the effect of varying the MAF threshold used to define rare variants (0.005, 0.01, 0.05) on the type I error and power. We found that the type I error stayed consistent for the methods not including common variants and for the CMC method. For LASSO regression, the inflation seen in the type I error appears to decrease as the MAF cutoff increases. This is likely because fewer SNPs are defined as common variants as the MAF cutoff increases, and thus fewer common SNPs are undergoing selection by means of LASSO regression, decreasing the type I error. The changes in power because of the different MAF cutoffs are dependent on the particular characteristics of each gene. The power varies between MAF cutoffs only if the MAF of one or more risk SNPs within the gene is close to the threshold. Because the most appropriate MAF cutoff varies for each gene, a method that uses variable MAF thresholding may be more powerful (see description of Price et al.’s method later in this section).

A common underlying assumption for the indicator and count methods is that the rare variants within a trait-associated gene have effects in the same direction. An even stronger assumption—that all rare variants within a gene that are associated with a trait have the same effect size—is also required for the count method. Although these assumptions are usually not satisfied, the GAW17 data were simulated to fulfill the first assumption in that all minor alleles are associated with an increase in Q1. The second assumption of equal effect size was not met by most of the simulated risk gene regions. The data-adaptive method also assumes that all causal rare variants within a gene have the same effect size, but unlike the simple count method, the data-adaptive sum test allows the direction of the effect to be different. In real data, the data-adaptive approach requires permutation to retain the appropriate type I error. Using permutation increases the computation expense but is necessary because the method biases the data in favor of association before the analysis. Thus, for the data-adaptive method, there is a trade-off between flexibility and computational expense. As discussed in the Methods section, we use an empirically derived significance threshold calculated from the null phenotype, Q4, to find the power for each method. Given the simulated null distribution, using the empirical significance threshold is computationally fast and efficient compared to permutation. In real data, where a null distribution is not available, permutation is likely the better alternative.

In this study, we compared several published approaches and some modifications to analyze rare variants; however, there are other approaches available that we did not use in our comparisons. Capanu et al. [7] applied pseudo-likelihood and Bayesian approaches to conduct hierarchical modeling to leverage the collective evidence from rare variants. Madsen and Browning [8] used a weighted-sum statistic to jointly analyze a group of rare variants. These methods were developed for case-control data and need modifications before they can be applied to quantitative traits. More recently, Price et al. [9] developed a pooled association test, which uses a variable threshold to determine the MAF cutoff to define rare variants and also incorporates biological information. King et al. [10] developed an evolutionary mixed model for pooled association testing; this approach uses population genetic theory to provide prior information on effect sizes and a rare variant pooling strategy. Hoffmann et al. [11] proposed a step-up collapsing approach that determines an optimal grouping of rare variants analytically without relying on prior information.

Here, we have compared approaches that collapse the genotypes for all rare variants within a predefined gene region. An obvious expansion of these methods would be to use existing biological information, such as the functionality of each marker, to choose a subset of rare variants or to weight the rare variants. In addition, other information, such as rare variant transmission through a pedigree or linkage analysis, might also help to guide the weighting and grouping of rare variants within a gene region.

## Conclusions

The three methods compared here for collapsing rare variants—indicator, count, and data-adaptive methods—performed similarly in this simulation design in which all rare risk alleles were simulated to be in the same direction. Incorporating common variants in order to detect a gene region was useful when there were common causal variants within the gene. And finally, using a selection method, such as LASSO regression, to determine which common variants to include in the analysis was useful when there was more noise than signal as a result of common SNPs in the gene.

## Competing interests

The authors declare that there are no competing interests.

## Authors’ contributions

HC carried out the indicator, count, and data-adaptive analyses. AEH carried out the LASSO analysis, and coordinated the study. YC carried out the CMC analysis. LAC and JD helped to conceive the study. CTL conceived the study. HC, AEH, YC, JD, and CTL participated in the design of the study and helped draft the manuscript. All authors read and approved the final manuscript.
